# Thermal plasticity in *Drosophila melanogaster*: A comparison of geographic populations

**DOI:** 10.1186/1471-2148-6-67

**Published:** 2006-08-30

**Authors:** Vincenzo Trotta, Federico CF Calboli, Marcello Ziosi, Daniela Guerra, Maria C Pezzoli, Jean R David, Sandro Cavicchi

**Affiliations:** 1 Alma Mater Studiorum, Università di Bologna, Dipartimento di Biologia Evoluzionistica Sperimentale, via Selmi 3, 40126 Bologna, Italy; 2 Department of Epidemiology and Public Health, Imperial College, St Mary's Campus Norfolk Place, London W2 1PG, UK; 3 CNRS, Laboratoire Populations, Génétique et Evolution, 91198 – Gif sur Yvette Cedex, France

## Abstract

**Background:**

Populations of *Drosophila melanogaster *show differences in many morphometrical traits according to their geographic origin. Despite the widespread occurrence of these differences in more than one *Drosophila *species, the actual selective mechanisms controlling the genetic basis of such variation are not fully understood. Thermal selection is considered to be the most likely cause explaining these differences.

**Results:**

In our work, we investigated several life history traits (body size, duration of development, preadult survival, longevity and productivity) in two tropical and two temperate natural populations of *D. melanogaster *recently collected, and in a temperate population maintained for twelve years at the constant temperature of 18°C in the laboratory. In order to characterise the plasticity of these life history traits, the populations were grown at 12, 18, 28 and 31.2°C. Productivity was the fitness trait that showed clearly adaptive differences between latitudinal populations: tropical flies did better in the heat but worse in the cold environments with respect to temperate flies. Differences for the plasticity of other life history traits investigated between tropical and temperate populations were also found. The differences were particularly evident at stressful temperatures (12 and 31.2°C).

**Conclusion:**

Our results evidence a better cold tolerance in temperate populations that seems to have been evolved during the colonisation of temperate countries by *D. melanogaster *Afrotropical ancestors, and support the hypothesis of an adaptive response of plasticity to the experienced environment.

## Background

In any species, the intrinsic rate of natural increase (the '*r*' parameter) integrates several characteristics of the life cycle, which are generally described as life history traits [1-3]. Variations in these traits are directly related to demographic changes and are thus related to individual fitness [4]. However, all natural populations live in avariable environment, and life history traits are strongly influenced by such variations. In this sense, the response curves of fitness traits to the environmental changes need to be investigated. Environmental variations (e. g. temperature) may beconsidered as benign if their mean value is close to the functional optimum of the trait. Greater variations may be deleterious and are considered stressful [5-7]. A major problem in ecological genetics is therefore to understand the evolutionary responses to stressful variations. If a given stress is really exceptional in its intensity, it is likely to result in the extinctionof a population without eliciting an adaptive response. Milder and more repetitive stresses, on the other hand, are expected to induce an adaptive change, and the role of stress in shaping the genetic architecture of life history traits is a regular problem in evolutionary biology [5,6].

A convenient way to analyse such adaptive responses is to compare populations of the same species living under different climates. We expect that, under tropical conditions, heat stresses will be frequent. Reciprocally, cold stresses will be frequent in a temperate country [8]. *Drosophila melanogaster *may appear as an ideal model organism for investigating evolutionary responses to thermal selection. Its whole thermal range goes from 11 to 32°C, but both extremes are highly stressful, since they do not permit the development of successive generations [9,10]. The occurrence of latitudinal clines on different continents for developmental time [11,12], egg size [13] and body size [12,14-19] implicates a selective role of climate differences. In particular, it is likely that temperature promote the evolution of clinal size differences since its environmental impact varies in a predicted way along clines; nevertheless, many other factors such as day length, number of generations for breeding season, rainfalls are correlated with temperature variations in nature. Long term selections in laboratory cultures have resulted in divergent body sizes at different temperatures, in a way similar to that observed in natural populations [20-23]. As suggested by Santos *et al*. [24], larval crowding may also play a part in the establishment of body size differences, since natural or laboratory occurring differences in larval crowding involve correlated changes in life history traits during adaptation [25,26]. However, the relationship between temperature and larval density has never been addressed.

Finally, because ancestral populations of *D. melanogaster *are found in the Afrotropical region [14], it is possible to infer the direction of evolution, from tropical to temperate. For a deeper analysis of climatic adaptation, we need to investigate traits which are directly related to fitness, that is life history traits. Until recently, relatively few relevant investigations have been carried out, presumably due to the fact that these traits are difficult to be accurately measured in laboratory conditions. Evidence for climatic adaptation comes from clinal patterns in traits [8], nevertheless it is not known if a certain trait is the direct target or a byproduct of natural selection and if temperature is the main selective factor in nature. Two different strategies are possible to verify if a life history trait and its variation are related to climate. The first is to collect numerous natural populations along a thermal gradient and demonstrate the existence of a latitudinal cline in the trait under study [8,11,12]. The second strategy is to comparepopulations from the two ends of a cline, and make a deeper analysis by considering either the phenotypic plasticity of each population [27-29], or the genetic architecture of several traits [30].

Since our aim is to investigatelife history traits, we have chosen the second strategy. We consider two tropical populations of *D. melanogaster*, adapted to a warm environment, and two temperate ones, adapted to a much colder climate and especially to cold winters. Several life history traits have been measured, that is body size, viability, developmental time to adulthood, progeny production and longevity. Since these populations lived in different thermal environments, we used in each case 4 different experimental temperatures, either benign or stressful. To test if the selective history of the populations was changed by laboratory rearing conditions (e. g. inbreeding or genetic drift), we compared the results of these recently collected populations to those of a long-adapted laboratory strain from a temperate origin, kept at a constant temperature of 18°C for 12 years. Adaptive responses have been observed for developmental rate, progeny production and body size, but not for longevity; results for viability were ambiguous.

## Results

### Wing area

**Figure 1:**
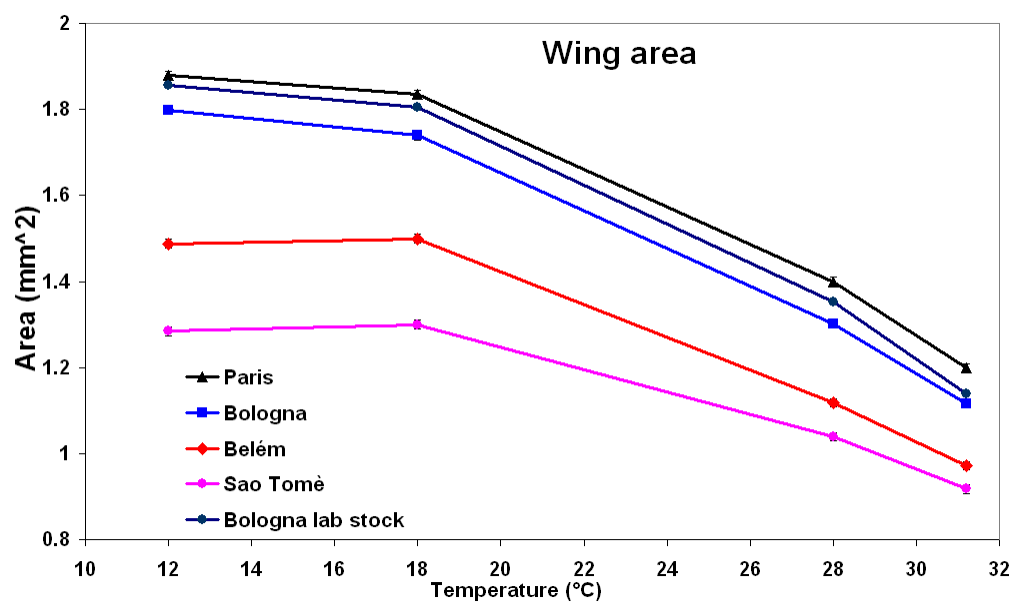
**Wing area**. Mean wing area in mm^2 ^(± standard error) of the five populations of *D. melanogaster *over the thermal range.

A number of differences between temperatures and populations was expected and was found (Fig. 1 and table 1). Temperate populations were bigger than the tropical ones over the whole thermal range (location, *P *< 0.001) in spite of significant differences between populations within location (*P *< 0.001), and flies reared at colder temperatures were bigger than flies reared at warmer temperatures for all populations (*P *< 0.001). The same results were obtained when the data of the Bologna lab population were omitted from the analysis (data not shown).

**Table 1:** Results of the ANOVAs (mixed model) on wing area, viability (performed on the square root of the arcsine of the percentage value) and developmental time. The populations were grouped, with respect to their origin (Location), in temperate (Paris, Bologna and Bologna thermal stock 18°C) and tropical (Belém and Saõ Tomè). Location and temperature are fixed effects, population is nested within location within temperature.

		WING AREA		VIABILITY		DEVELOPMENTAL TIME	
				
*Source of variation*	*df*	MS	*F*	MS	*F*	MS	*F*
Location	1	5.3095	103.9 ***	0.0026	0.0057	14.53	3.09 †
Temperature	3	4.31	84.3 ***	1.294	2.81 †	17901	3775 ***
Location × temperature	3	0.1539	3.01 †	0.274	0.6	13.67	2.88 †
Population within location and temperature	12/12/11	0.0511	33.9 ***	0.46	69.4 ***	4.7	8.11 ***
Residuals	180/175/170	0.0015		0.0067		0.585	

† 0.10 <*P *< 0.05; ***P < 0.001; *df*, degrees of freedom; MS, mean square; *F*, variance ratio.

The shapes of the response curves to growth temperature (Fig. 1), or reaction norms, were adjusted to polynomials of degree 2, allowing the calculation of the temperature of maximum size for each population [31]. The temperature of maximum size is higher in the tropical populations (14.4 ± 0.5°C for Belém and 15.1 ± 1°C for Saõ Tomè) than in the temperate ones (12.8 ± 0.6°C for Paris, 11.7 ± 1.1°C for Bologna and 12.2 ± 1°C for the Bologna laboratory population).

### Viability

**Figure 2:**
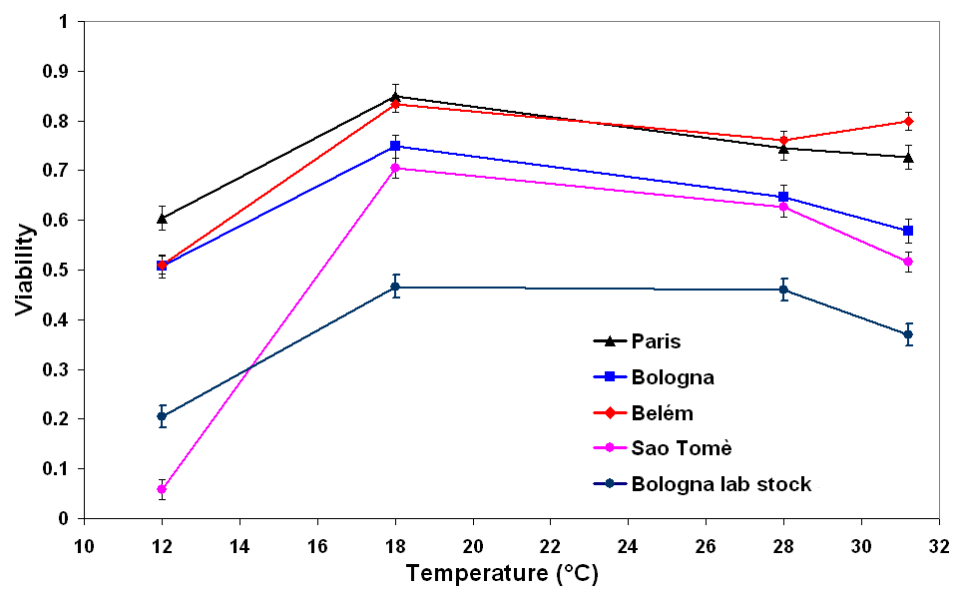
**Viability**. Mean **v**iability ( ± standard error) of the five populations of *D. melanogaster *over the thermal range.

At 18°C, a temperature close to the optimum for *D. melanogaster *[32], all the populations showed the highest viability (Fig. 2). A drop in viability was recorded at 12°C (particularly for the population from Saõ Tomè); this drop was less evident at 31.2°C. At 12°C the viability of the Bologna lab stock was estimated from five vials. ANOVA performed on the square root of the arcsine percentage of viability (Table 1) revealed significant differences among populations (*P *< 0.001) and slightly significant differences among temperatures (0.10 <*P *< 0.05). No significant "location by temperature" interaction or differences between locations were found.

The Bologna lab stock, compared to the other populations, showed the lowest viability over the whole thermal range, but the conclusions of the ANOVA did not change when this population was omitted from the analysis.

### Developmental time

**Figure 3:**
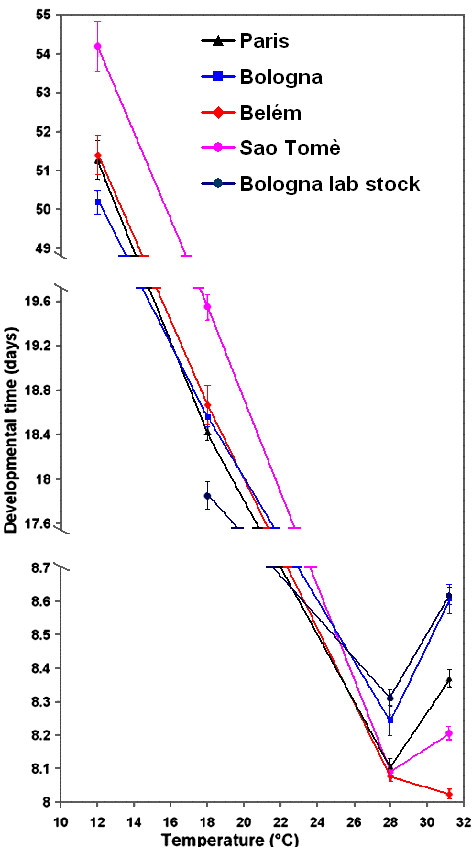
**Developmental time**. Mean developmental time in days ( ± standard error) of the five populations of *D. melanogaster *over the thermal range.

Fig. 3 shows the mean developmental time of the five populations (except for the Bologna lab stock at the experimental temperature of 12°C).

A mixed model ANOVA (table 1) gave significant differences among populations (within locations and temperatures) and among temperatures (*P *< 0.001 in both cases). Developmental time was shorter at 28°C and 31.2°C than at 18°C and 12°C. A general increase in developmental time was recorded for all but the Belém populations at 31.2°C when compared to 28°C, probably due to harmful effects of heat [8]. No significant differences between locations or significant "location by temperature" interaction were found for developmental time. Nevertheless, an increase in developmental time is observed at the most stressful temperature in agreement with the origin of the populations. The same results were obtained when the data of the Bologna lab population were omitted from the analysis (data not shown). Compared to the other populations, the Bologna lab stock showed the lower developmental time at 18°C, the temperature it was maintained to for twelve years.

### Longevity

For the analysis of longevity, as well as for the productivity, all flies were grown in the same thermal environment (25°C) and emerged adults were transferred to the four experimental temperatures.

**Figure 4:**
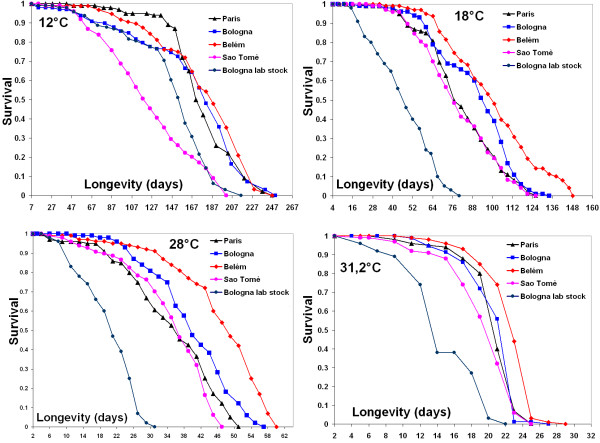
**Longevity**. Caplan-Meier survival plot of females of the five populations of *D. melanogaster *at each experimental temperature. Data are based on a total of about 100 individuals per population.

Survival plots for females are shown in Fig. 4. A Cox proportional hazard model was used and detected significant differences among populations for each temperature (with the only exception for females at 12°C, table 2). Surprisingly, for both males and females, the differences found did not match the tropical/temperate division. The Bologna lab stock is the shortest lived population, even in the thermal environment in which it was selected. Among the natural populations, in general the two longest lived populations are Belém and Bologna, and the two shortest lived are Paris and Saõ Tomè.

**Table 2:** Results of Cox proportional hazard analysis for survivorship of the populations at the four experimental temperatures. A) females; B) males. Population is nested within location (fixed effect).

A) Females									
		12°C		18°C		28°C		31.2°C	
					
*Source of variation*	*df*	MS	*F*	MS	*F*	MS	*F*	MS	*F*

Location	1	22.8	1.79	38.3	0.52	60.1	0.61	50.4	0.77
Population within location	3	12.7	1.23	73.9	7.4 ***	98.2	10 ***	65.2	6.5 ***
Residuals	479/491/491/489	10.3		9.98		9.8		10	

B) Males									

*Source of variation*	*df*	MS	*F*	MS	*F*	MS	*F*	MS	*F*
Location	1	0.4	0.01	24.8	0.34	71.1	0.59	56.3	0.66
Population within location	3	39	3.8 *	72.2	7.2 ***	119.7	12.4 ***	84.9	8.6 ***
Residuals	495/483/490/492	10.3		10		9.6		9.88	

* *P *< 0.05; ***P < 0.001; *df*, degrees of freedom; MS, mean square; *F*, variance ratio.

Longevity seems to be population-specific but not correlated with geographical origin, even when the Bologna lab population was omitted from the analysis. At extreme cold the tropical population from Saõ Tomè showed a very short life span, suggesting a specific cold sensitivity.

### Productivity

**Figure 5:**
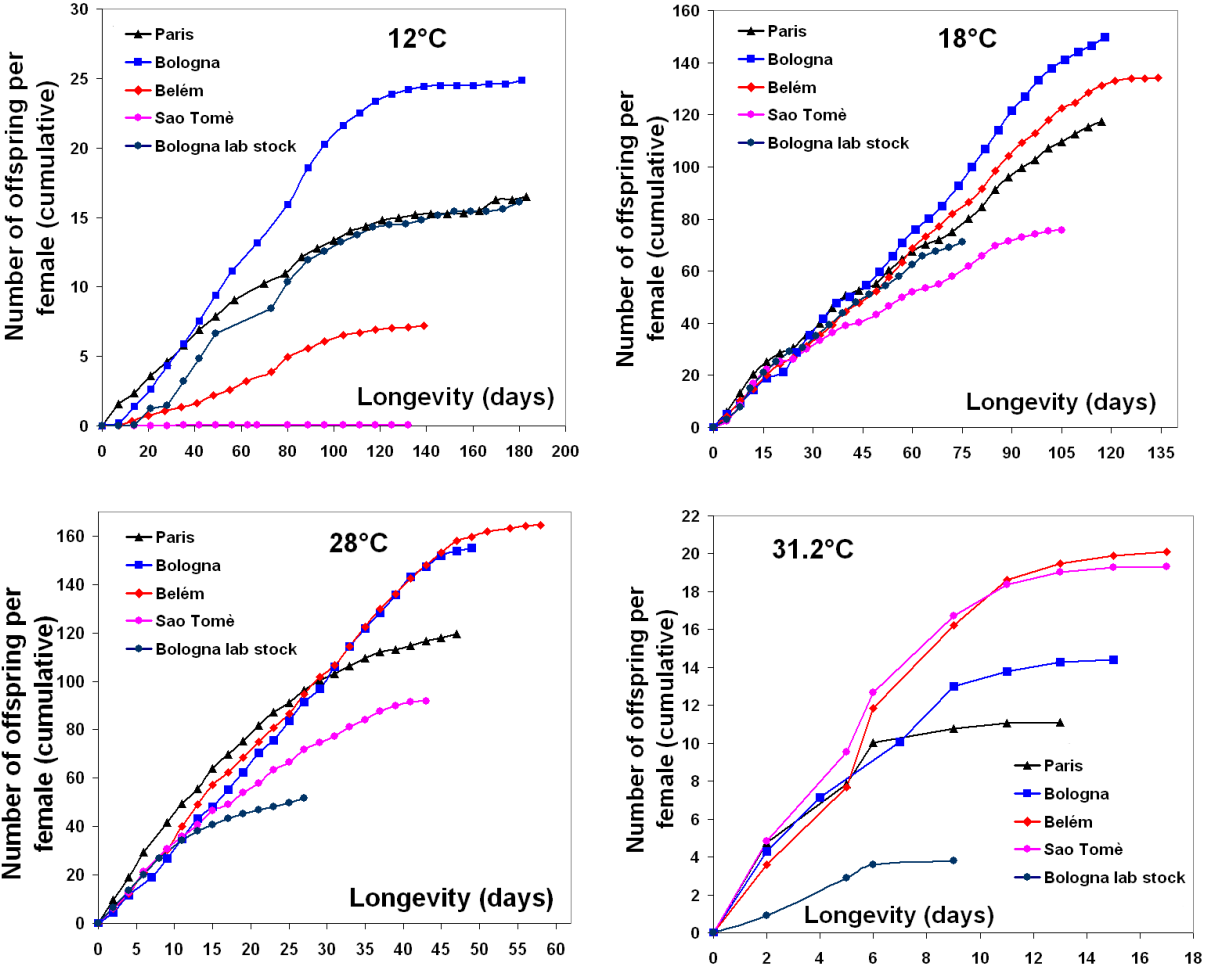
**Cumulative productivity**. Cumulative productivity per female over time of the five populations of *D. melanogaster *at each experimental temperature.

We used the cumulative offspring production as an indication of the fitness of the populations (Fig. 5). The best fit for the cumulative productivity over time was given by two degree polynomials passing through the origin (R^2 ^> 0.96 in all the cases). An ANCOVA was then performed at each temperature in order to identify differences in slopes between polynomials that are indicative of differences in reproductive rates between locations and between populations. The interaction between polynomial regressions and populations was significant for each temperature (*P *< 0.001 in all cases, table 3). A slightly significant difference between locations was found only at 31.2°C (*P *= 0.056); only the last difference became more pronounced when the Bologna lab population was omitted from the analysis (*P *= 0.014). Because at 12°C the population from Saõ Tomè had no offspring production, it was omitted from the analysis and in the ANCOVA performed at this temperature the factor location is lacking.

**Table 3:** Results of ANCOVAs on the cumulative productivity over time of the populations at the four experimental temperatures. The cumulative productivity over time was approximated by two degree polynomials passing through the origin. Location is a fixed effect, population is nested within location.

a) ANCOVA at 12°C. The population from Saõ Tomè had no offspring production and was omitted from the analysis.							
*Source of variation*	*df*	MS	*F*				

Polynomial regression	2	1888	3514 ***				
Polynomial regression X population	6	70	130 ***				
Residuals	83	0.5					

b) ANCOVA at 18°C, 28°C and 31.2°C.							

		18°C		28°C		31.2°C	

*Source of variation*	*df*	MS	*F*	MS	*F*	MS	*F*

Polynomial regression	2	101664	13899***	113109	16759***	660	1412***
Polynomial regression X location	2	592	0.597	17	0.01	35.9	4.8 †
Polynomial regression X population within location	6	991	135.5***	1631	241.6***	7.5	16 ***
Residuals	126/100/24	7		7		0.5	

† 0.10 <*P *< 0.05; ***P < 0.001; *df*, degrees of freedom; MS, mean square; *F*, variance ratio

The natural populations from temperate regions (Bologna and Paris) produced more offspring at the extreme cold compared to both tropical populations (at which Saõ Tomè had no offspring production). This trend was reversed at the extreme hot. The productivity of the Bologna lab stock was similar to the other two temperate populations at 12°C and 18°C. At hot temperatures (28°C and 31.2°C) the Bologna lab stock showed the lowest productivity. At permissive temperatures the productivity of the five populations was higher compared to both extremes temperatures, but in these cases the differences between tropical and temperate populations did not show a clear pattern.

**Figure 6:**
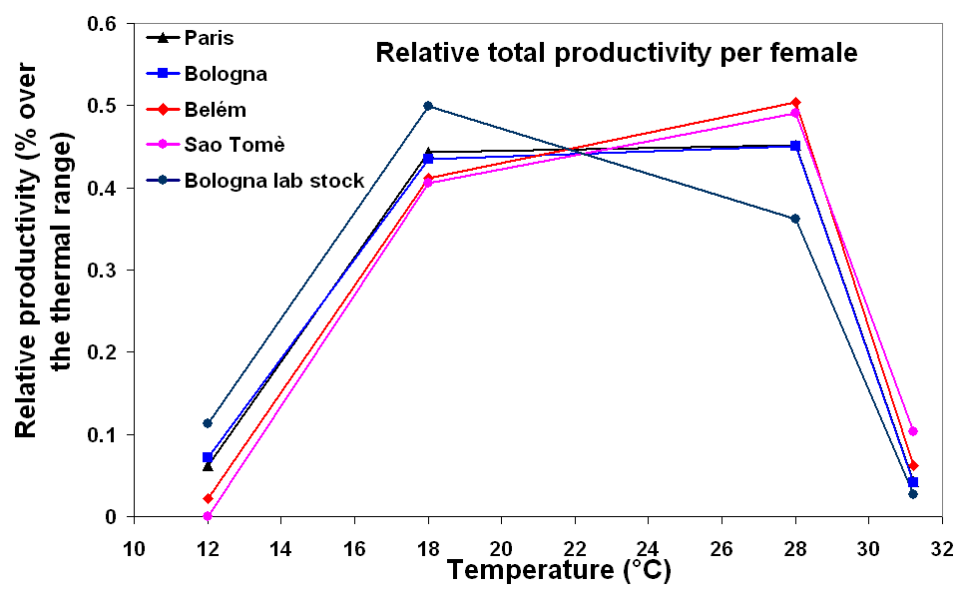
**Relative total productivity**. Relative productivity per female (ratio between the mean number of offspring at a given temperature and the total number of offspring over the whole thermal range within each population) of the five populations of *D. melanogaster*.

For a better comparison of the populations, we standardised the performance curves on the total productivity at the four temperatures within each population (Fig. 6). The ANOVA (table 4) performed on these standardised values, grouping the populations in temperate and tropical, gave significant differences between temperatures and a significant "location by temperature" interaction (*P *< 0.01 in both cases). Tropical populations showed a shift of the curve toward warm temperatures whereas the natural temperate populations (Paris and Bologna) showed a constant performance at intermediate temperatures (18°C and 28°C), with higher productivity at extreme cold but lower at extreme hot than the tropical ones. The Bologna lab stock showed a further shift of the curve toward cold temperatures.

**Table 4:** A two-way ANOVA on the relative productivity per female with location and temperature as fixed effects.

*Source of variation*	*df*	MS	*F*
Location^+^	1	0	0
Temperature	3	0.255	290 ***
Location X temperature	3	0.00623	7.08 **
Residuals	12	0.00088	

^+ ^The between Location variance is 0 as we imposed the same productivity to each population.

**P < 0.01; ***P < 0.001; *df*, degree of freedom; MS, mean square; *F*, variance ratio.

## Discussion

*D. melanogaster *is known to exhibit numerous genetic differences between tropical and temperate populations [14,18,33]. Here we have compared various life history traits in four geographic populations of *D. melanogaster*, two tropical and two temperate, and one laboratory stock of temperate origin (Bologna). The cline ends populations in study were analysed one or three years after collection, while the laboratory stock had been subjected to a constant thermal regime of 18°C for twelve years. The laboratory stock showed a higher size than the Bologna population sampled in the same place in 2002, probably due to an effect of thermal selection [21-23]. In addition to that, two life history traits, viability and longevity, showed a decrease over the whole thermal range and, as a consequence of a short lifespan, also the total productivity was lowered but with a similar trend respect to the other two temperate populations. Though the data are referred to one line only, they suggest that inbreeding depression is a possible cause, since it can affect most fitness components [34-38].

It is known that laboratory adaptation may alter some life history traits [39]. Nevertheless, on the basis of our observations, we found that some adaptive differences in life history traits among natural populations persist in spite of laboratory adaptation [40] or inbreeding effects; other life history traits change only their mean values and not their plastic response over a thermal range. This has also been verified several times for morphometrical traits [12,41].

In general, our results show that some life history traits are indicative of temperature selection in tropical and temperate populations while others are not.

Adult longevity was a trait for which we did not find a significant difference between tropical and temperate flies over the whole thermal range. Longevity in nature is known to be quite short depending on season and place [8,42], and what is measured in the lab might be unrelated to fitness in the wild.

For body size traits our data confirmed the expected bigger size of temperate populations [33,41].

What is more interesting is that the phenotypic plasticity seems also to have changed. We known that *D. melanogaster *is a species of Afrotropical origin, and that climatic adaptation has occurred from tropical towards temperate climates [43]. During the colonisation of temperate continents, adaptation has not only produced bigger flies, but also a shift in the phenotypic plasticity of the wing: the maximum size is observed at a lower temperature in temperate populations. Such a change in the shape of the reaction norms is an interesting confirmation and generalisation of an observation which was previously made on two populations only, from France and West Indies [29].

To our knowledge, it is the first time that duration of development was investigated over a broad thermal range in different populations. A clear adaptive difference has been observed at the stressful temperature of 31.2°C: tropical populations developed faster than temperate ones, though this difference was not clearly observed over the whole thermal range. The natural selection a population was subjected to seems to be weakly reflected in developmental time, since an increase in the duration of development is observed only at one stressful developmental temperature according to the climate of origin of a population. This result is in agreement with a previous comparison between Afrotropical and European populations that failed to highlight any significant difference in the duration of development [44].

As noted before, the Bologna lab stock showed the lowest viabilityover the whole thermal range. The tropical populations showed a relative drop in viability at extreme cold (particularly evident for Saõ Tomè). However, a clear geographic pattern was not observed. Population viability (like longevity) seems therefore to be independent from the experienced environments, indicating that this trait (and its plasticity) is population-specific but not related to the selective history of a population unless a stressful condition is encountered.

Finally, for offspring production, we also observed an interaction between the reaction norms, especially when data from each population were standardised for the same overall mean (Fig. 6). Again, the functional interpretation is straightforward: temperate flies do better in the cold but worse in the heat than tropical ones. A very interesting observation is that adults from Saõ Tomè, when transferred to 12°C, failed to produce any offspring, although their average longevity exceeded 100 days.

## Conclusion

Most life history traits in *Drosophila melanogaster *seem to have evolved, in an adaptive way, during the colonisation of temperate continents from Afrotropical ancestors. Cold is in fact considered the most stressful condition experienced by *D. melanogaster *in temperate areas (as evidenced by a marked reduction in population size) so the adaptation to winter conditions leads to an increase in overall fitness in temperate populations under a cold environment [8].

The response to thermal extremes is clearly an indication of thermal adaptation in natural populations, but which is the role of thermal stress in shaping the adaptation to a given environment? A possible answer is that adaptation to an "average" thermal environment automatically create a level of preadaptation to a thermal stress that is close to the mean temperature normally experienced. As a consequence, even the plasticity curve of some life history traits of population can evolve, being shifted toward a new optimum. While this hypothesis cannot be entirely discarded, the increased cold tolerance found in temperate populations can be explained as an adaptive response to the cold stress. What was really not expected was the fact that those populations lost part of their heat tolerance, being no more subjected to heat stress.

The interpretation of this apparent trade-off is not obvious. One possibility is that maintaining a superfluous heat tolerance have a cost, which explains a backward selection. Another possibility would be a negative pleiotropy: alleles increasing cold tolerance could also exhibit a lesser heat tolerance. A last problem is the identity of the genes which, acting on very different traits (body size, duration of development, progeny production), altogether contribute to the climatic adaptation of the populations. We hope that, in the future, specific investigations will be undertaken for unraveling the nature of such quantitative trait *loci*.

## Methods

### Populations

Five different populations of *Drosophila melanogaster *were used in the present work, two tropical and three temperate. One temperate population came from Draveil, near Paris (48°44N, collected in 2002), where the average temperature oscillates from 3°C in January to 20°C in July; another temperate population came from Bologna (44°30N, collected in 2002) where the average temperature oscillates from 4°C in January to 24°C in July. Of the two tropical populations, one came from Belém, Brazil (1°27S, collected in 2002) and the other from Saõ Tomè (0°20N, collected in 2000) where the average temperature is around 25–28°C and quite stable all over the year. These four strains were founded from 10–15 pairs of wild-collected flies and kept in mass culture on standard medium at 20°C until April 2003, when the experiment started.

The various populations experienced different periods of laboratory rearing, ranging from six months to three years. Though it was found that morphological traits do not change in a few years [41], laboratory adaptation may be a potential problem in most evolutionary studies [39], since it seems to change preadult development, fecundity, survival rate of females [45] and to decrease stress resistance [46]. On the other side, Matos and Avelar [40] claim that laboratory adaptation is not a problem for interpretation of evolutionary studies since any laboratory regime can give information on the evolutionary potentialities of a population. Laboratory rearing would produce inbreeding and drift that could affect some life history traits, causing reduced viability, lower fecundity, decreased mating success [34-38]. For these reasons we introduced in the experiment as a control a temperate population derived from a natural population sampled in Bologna on October 1991. The offspring of thirty fertilised females was kept at the constant temperature of 18°C for twelve years. Each generation was reared on a standard medium in 250 ml bottles, for a total of 10 bottles. On emergence, thirty pairs were collected and allowed to lay eggs for a few days. This population can be considered a laboratory population subjected to constant temperature adaptation [47].

### Experimental procedures

To avoid maternal effects, flies were allowed to oviposit for a day at a constant temperature of 25°C and at an optimal density (about 100 individuals in 60 ml vials containing 10 ml of food, density that assures the achievement of the maximum body size) for one generation. The emerging flies were transferred in bottles with an egg laying dish containing apple juice medium smeared with abundant yeast. To collect synchronised eggs, several hundred females were allowed to oviposit for two days at 25°C, changing the egg laying dishes every day; on the third day dishes were changed three times every 2h, then the egg collection started. 70 eggs were counted and transferred in 60 ml vials containing 10 ml of food. Vials were allocated to two groups. The first group, consisting of forty vials, was maintained until adult emergence at the different constant temperatures of 12°C, 18°C, 28°C and 31.2°C (ten vials for each temperature). Individuals from this first group of vials were used to measure viability, developmental time to adulthood and wing area. The second group, consisting of about twenty-five vials, was left at 25°C until emergence. One day after, these flies were distributed in forty vials (ten males and ten females each) and transferred to the four experimental temperatures (ten vials for each temperature). These vials were used to measure longevity and total productivity over time. The same protocol was used for all the populations.

### Wing area

From the first group of vials, five females per vial were randomly chosen, for a total of 50 individuals for each temperature. Right wings were dissected, dehydrated in ethanol and mounted on glasses in lactic acid/ethanol (6:5). Wing images were captured using a Zeiss optical microscope mounting an Axiocam digital camera. For each wing the total area was taken; all the measures are in mm^2^. The analysis was performed pooling individual values within vial (five flies), since the "between vials within temperature and population" variance was never found significant (data not shown).

### Developmental time and viability

Developmental time was measured as the days elapsed between egg laying and adult emergence. At 18°C, flies were collected twice a day; at 28°C and 31.2°C flies were collected three times a day. At 12°C they were collected once a day. These flies were also used to investigate viability and wing area. The developmental time was calculated as the grand sum of the number of emerged adults (n_i_) multiplied by the time at which they eclosed (t_i_, in days), all divided by the total number of emerged flies (N), that is = (Σ n_i _* t_i_)/N. The mean of the ten vials was taken as an estimate of the developmental time of a given population at a given temperature. Unfortunately, we have not the estimate of the developmental time of the laboratory line sampled in Bologna in 1991 (the lab stock) at the experimental temperature of 12°C.

Viability was estimated as the percentage of flies emerged from the counted eggs.

### Longevity

For each population and temperature ten vials containing food were established with ten males and ten females each. At 12°C the ten pairs were transferred to fresh vials every week, at 18°C every four days and at 28°C and 31.2°C every two days, to compensate for the increased rate of living at the higher temperatures [48]. Mortality was scored every time flies were transferred to fresh medium. In order to keep the crowding and the sex ratio roughly constant within vials, flies from all vials were pooled at every transfer and reallocated to fresh medium at a constant crowding of 10 males and 10 females per vial (or the best approssimation in case the number of flies was not a multiple of 10).

For each population, a Cox proportional hazard model was used to test for differences between sexes, temperatures and their interaction. We found differences between temperatures and sexes (females are a little more long-lived than males) but no interaction between sexes and temperatures was found (data not shown). Data were then analysed separately for sexes and temperatures and data from females were graphically represented with a Caplan-Meier survival plot.

### Productivity

The flies used for this assay were the same used for the longevity assay; therefore, a constant number of flies of the same age was kept in all vials in order to avoid larval crowding effects on larval viability. We counted the number of adults emerging from each vial after the parents were transferred to new vials. Because the exact number of females was known for each vial, it was possible to have an index of productivity, dividing the number of emerging flies by the number of females.

Productivity analysis used the cumulative offspring production per female at each temperature. Moreover, the use of cumulative offspring production allows to visualise which population had the maximum offspring production and the time needed to reach it.

### Statistical analysis

Wing area, viability and developmental time were analysed by mixed model ANOVAs in which location (temperate and tropical populations) and temperature were main fixed effects and population was nested within location and temperature. These analyses were used to test for population differences, using an error term that includes the variation among vials; temperature, location and their interaction were tested against the between populations variance. For the longevity analysis, Cox proportional hazard regression models were used to test for differences between locations and populations (nested within locations); sexes and temperatures were analysed separately. The cumulative productivities at each temperature were analysed by ANCOVAs to test for differences between locations and populations within locations. The total productivity was also standardised across temperatures within each population and analysed with a two-way ANOVA with location and temperature as fixed effect. All the analyses in this work were performed with R 2.2.0 [49].

## Authors' contributions

VT designed and performed the study, carried out the preliminary statistical analyses and drafted the manuscript. FCFC carried out the statistical analyses. MZ, DG and MCP made substantial contributions to the manuscript. JRD provided the flies, helped to conceive the study and drafted the final manuscript. SC conceived the approach and drafted the final manuscript. All authors read and approved the final manuscript.
